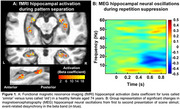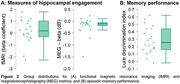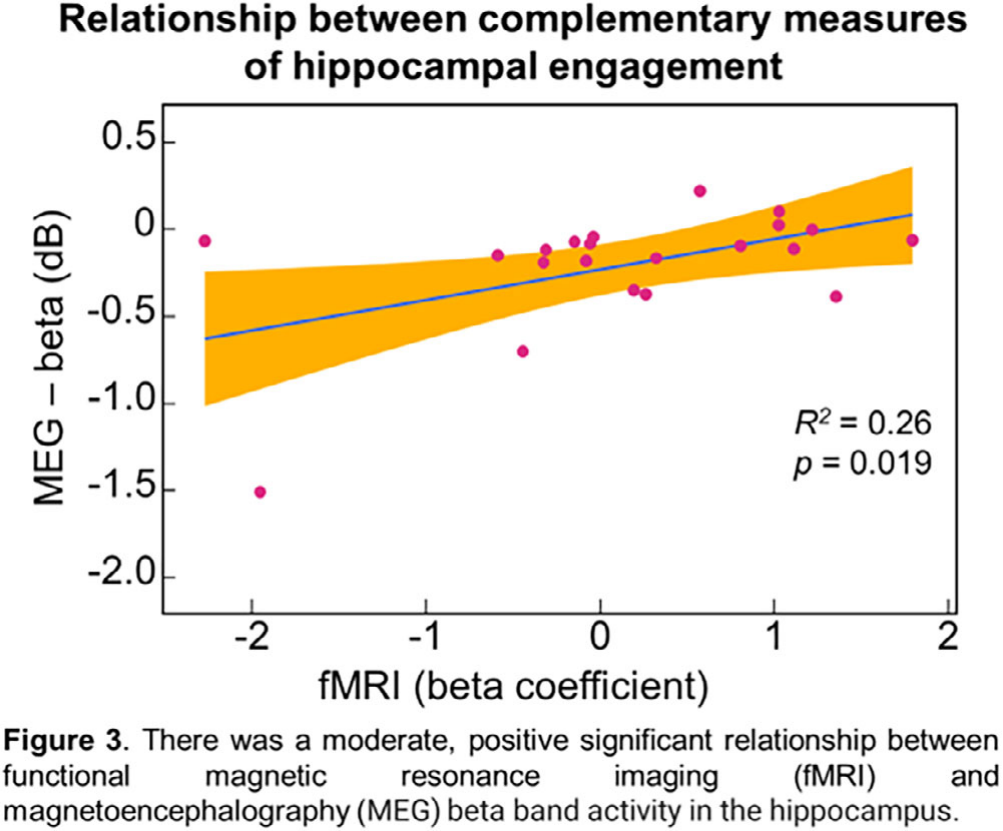# Multimodal functional neuroimaging of hippocampal engagement in cognitively normal older individuals

**DOI:** 10.1002/alz70856_102965

**Published:** 2025-12-25

**Authors:** Kevin Grant Solar, Melisa Gumus, Sriranga Kashyap, Nicolas Deom, Ljubica Zotovic, Kamil Uludag, Krista L Lanctôt, Sandra E. Black, Luis Garcia Dominguez, Richard Wennberg, Mary Pat McAndrews

**Affiliations:** ^1^ Krembil Brain Institute, Toronto Western Hospital, University Health Network, Toronto, ON, Canada; ^2^ University of Toronto, Toronto, ON, Canada; ^3^ Sunnybrook Research Institute, Toronto, ON, Canada; ^4^ Krembil Brain Institute, Sunnybrook Research Institute, Toronto, ON, Canada; ^5^ Sunnybrook Research Institute, University of Toronto, Toronto, ON, Canada; ^6^ Toronto Western Hospital, University Health Network, Toronto, ON, Canada; ^7^ Canadian Concussion Centre, Toronto Western Hospital, University Health Network, Toronto, ON, Canada

## Abstract

**Background:**

Hippocampal hyperactivation in fMRI has been observed in individuals at risk for Alzheimer's Disease (AD), both those with normal cognition (NC) and those with mild cognitive impairment (MCI). This hyperactivity may serve as a biomarker for identifying at‐risk individuals, as well as a promoter of tau pathology, thus offering the potential to delay or avert cognitive decline through early interventions. Multimodal neuroimaging approaches are essential to better understand hippocampal hyperactivity. fMRI provides indirect hemodynamic measures of neural activity with high spatial resolution, whereas magnetoencephalography (MEG) is a more direct indicator with high temporal resolution. We compared fMRI and MEG responses in older individuals, with no risk factors for AD, to examine the relationship between these measures of hippocampal episodic memory engagement and provide insights into hyperactivity mechanisms to inform future interventions.

**Methods:**

21 participants (x̄_age_=70±7 [range=58‐81] years; 14 females) underwent fMRI (pattern separation task) and MEG (repetition suppression task). In the bilateral hippocampus, we calculated per subject beta coefficients for critical conditions (similar lures in fMRI, repeated stimuli in MEG). Additionally, we calculated the lure discrimination index (LDI) to indicate episodic memory performance, then utilized robust regression to test relationships between fMRI, MEG, and LDI.

**Results:**

Significant activation contrasts were identified for fMRI and MEG beta band activity (Figs 1 & 2). There was a significant, positive relationship between fMRI and MEG metrics of hippocampal engagement (*R^2^* = 0.26, *F*(1,19)=6.64, *p* = 0.019; Figure 3) and a positive trend between MEG beta band hippocampal activation and LDI (*R^2^* = 0.15, *F*(1,19)=3.27, *p* = 0.087).

**Conclusions:**

The importance of these results is multifaceted. First, we defined a direct neural read‐out of hippocampal activity in MEG that can index memory processes known to be affected in AD pathology. Second, we demonstrated the relationship between two complementary measures of hippocampal engagement (fMRI pattern separation, MEG repetition suppression) and their association to lure discrimination performance. Third, we established a normative distribution of both putative biomarkers that can be used for participant selection in future intervention trials.